# Antioxidant and Photoprotective Activities of 3,4-Dihydroxybenzoic Acid and (+)-Catechin, Identified from *Schima argentea* Extract, in UVB-Irradiated HaCaT Cells

**DOI:** 10.3390/antiox14020241

**Published:** 2025-02-19

**Authors:** Qi He, Yu-Pei Chen, Junhao Li, Hongtan Wu, Fangfang Chen, Mingyu Li, Chun Wu

**Affiliations:** 1 The School of Public Health, Fujian Medical University, Fuzhou 350122, China; 2220220176@fjmu.edu.cn (Q.H.); 2230220214@fjmu.edu.cn (M.L.); 2 The School of Public Health and Medical Technology, Xiamen Medical College, Xiamen 361023, China; wht@xmmc.edu.cn (H.W.); 200700010183@xmmc.edu.cn (F.C.); 3 Engineering Research Center of Natural Cosmeceuticals College of Fujian Province, Xiamen Medical College, Xiamen 361023, China; 4 Key Laboratory of Functional and Clinical Translational Medicine, Fujian Province University, Xiamen Medical College, Xiamen 361023, China; ljhhhh98@163.com

**Keywords:** *Schima argentea*, UVB-induced apoptosis, 3,4-Dihydroxybenzoic acid, (+)-Catechin, proteomic analysis

## Abstract

In traditional Chinese medicine, the root bark and leaves of *Schima argentea* are utilized to treat dysentery, parasitic infections, and digestive disorders. In this study, the n-butanol extract of *S. argentea* (NBA) exhibited potent antioxidant properties, protecting HaCaT cells from UVB-induced damage, and was abundant in phenolic and flavonoid compounds. Using UPLC-QTOF-MS analysis, several antioxidants within NBA were identified. Among these, 3,4-dihydroxybenzoic acid, (+)-catechin, and procyanidin B2 effectively reduced ROS levels after 1 h post-UVB treatment (225 mJ/cm^2^). Notably, all three compounds significantly decreased the phosphorylation of p38 and JNK in a dose-dependent manner. Additionally, the cell survival rate of these compounds was assessed after 12 h post-UVB treatment (225 mJ/cm^2^). Both 3,4-dihydroxybenzoic acid and (+)-catechin significantly prevented UVB-induced apoptosis in HaCaT cells, as evidenced by MTT, Hoechst, Calcein/PI staining, and flow cytometry analyses. Proteomic analysis revealed that 3,4-dihydroxybenzoic acid achieved photoprotection by downregulating c-Fos and Jun and modulating cell cycle proteins, while (+)-catechin promoted cell repair through the PI3K-Akt and Wnt signaling pathways. These results demonstrated that both compounds can directly absorb UVB, scavenge ROS, and provide cell photoprotection by modulating multiple signaling pathways. The n-butanol extract of *S. argentea* holds promising potential for future medical applications.

## 1. Introduction

Ultraviolet B (UVB) radiation induces DNA damage directly by forming cyclobutane pyrimidine dimers (CPDs), pyrimidone, and 6-4 photoproducts (6-4PPs) and indirectly by generating reactive oxygen species (ROS) [1,2,3]. ROS accumulation activates the Mitogen-Activated Protein Kinase (MAPK) pathway, enhancing activator protein-1 (AP-1) activity and upregulating matrix metalloproteinases (MMPs), which degrade collagen and contribute to skin photoaging [4]. UVB radiation also induces the tumor suppressor protein p53 through phosphorylation at Ser20 by c-Jun N-terminal kinase 1/2 (JNK1/2) or MAP Kinase-Activated Protein Kinase 2 (MAPKAPK-2). This modification prevents p53’s interaction with mouse double minute 2 (MDM2), stabilizing the protein to facilitate its response to DNA damage [5]. Additionally, UVB promotes cellular oncogene fos (c-Fos) expression via the p38 MAPK pathway, forming AP-1 complexes that modulate gene expression related to tumorigenesis [6]. It also activates the PI3K pathway, enhancing *c-fos* promoter activity and regulating cell survival and proliferation [7]. UVB triggers apoptosis by activating caspase-9 and caspase-3; cleaving PARP; downregulating anti-apoptotic proteins like myeloid cell leukemia-1 (Mcl-1), X-linked inhibitor of apoptosis protein (XIAP), and human inhibitor of apoptosis protein-1 (HIAP-1); and modulating protein kinase B (PKB) and protein kinase C (PKC) activity [8]. Overall, these mechanisms converge to induce cell apoptosis and mediate the cellular response to UVB radiation.

Plant-derived secondary metabolites, including phenolic compounds, carotenoids, and alkaloids, are crucial in mitigating the harmful effects of UV radiation [9]. Among these, phenolic compounds stand out for their potent antioxidant and anti-photodamage properties. They effectively scavenge ROS, inhibit the activity of MMPs, enhance DNA damage repair capabilities, and directly absorb UV rays, demonstrating a broad range of photoprotective effects [9,10]. Thus, these bioactive compounds have attracted much attention due to their multifaceted regulatory impact. For example, quercitrin and hyperoside, present in *Houttuynia cordata* extracts, modulate the MAPK and AKT signaling pathways, thereby alleviating UVB-induced oxidative stress and cellular apoptosis [11]. Similarly, 3,4,5-tri-O-caffeoylquinic acid (TCQA), found in the root extract of *Nymphoides peltate*, significantly reduces collagen degradation by activating the nuclear factor erythroid 2-related factor 2/antioxidant response element (Nrf2–ARE) signaling pathway and suppressing the MAPK/AP-1 and nuclear factor-kappa B (NF-κB) signaling pathways, highlighting its potential in anti-wrinkle therapy [12]. The seed extract of *Phaseolus angularis* L. exhibits exceptional antioxidant and anti-photoaging properties in UVB-exposed keratinocytes. It regulates the MAPK/AP-1 and Nrf2/ARE pathways, reducing collagen degradation by downregulating MMP-1 and MMP-3 expression while promoting skin repair through increased synthesis of procollagen type I and transforming growth factor-beta 1 (TGF-β1) [13]. Furthermore, the ethanol extract of *Breynia vitis-idaea* effectively scavenges free radicals, reduces UVB-induced ROS production, and inhibits the AP-1 pathway. The extract also exhibits notable anti-photoaging and anti-inflammatory effects by enhancing the synthesis of type I collagen (Col1A1) and suppressing the expression of MMP-3, MMP-9, and cyclooxygenase-2 (COX-2) [14].

*Schima superba* has garnered scientific interest for its potential medicinal properties. Studies have identified novel saponin compounds in the root bark of *S. superba* which exhibit a pronounced inhibitory effect on B16 melanoma cells, highlighting their potential in anti-melanoma therapies [15]. The stem bark extract of *S. superba* is particularly rich in saponins, including sasanquasaponin III, and has been found to possess robust antioxidant and anticandidal properties [16]. Additionally, kaempferol 3-rhamnoside, a flavonoid-derived secondary metabolite from *S. superba*, has been shown to significantly diminish glutamate release by targeting P/Q-type calcium channels and the calcium/calmodulin-dependent protein kinase II (CaMKII)/synapsin I pathway, thereby mitigating neuronal damage and protecting brain function [17]. Although research on *S. argentea* is limited, its close taxonomic relationship with *S. superba* suggests that it may share biological activities. *S. argentea* has been traditionally used in both medicine and agriculture [18]. Its bark and roots are employed in traditional Chinese medicine to treat dysentery, parasitic infections, and digestive disorders. Additionally, it finds applications in natural insecticides, veterinary medicine, and dermatology. In this study, we employed ultra-high-performance liquid chromatography–quadrupole time-of-flight mass spectrometry (UPLC-QTOF-MS) to characterize the chemical constituents of *S. argentea* extract. The antioxidant and photoprotective properties of the extract and its bioactive compounds, including 3,4-dihydroxybenzoic acid, (+)-catechin, and procyanidin B2, were comprehensively assessed through 2,2′-azino-bis(3-ethylbenzothiazoline-6-sulfonic acid) (ABTS), 2,2-diphenyl-1-picrylhydrazyl (DPPH), 3-(4,5-dimethylthiazol-2-yl)-2,5-diphenyltetrazolium bromide (MTT), and ROS detection assays. Furthermore, the impact of the extract’s bioactive compounds on UVB-induced apoptosis in human adult keratinocyte cell line (HaCaT) cells was investigated at the proteomic level to elucidate the molecular mechanisms underlying the antioxidant and photoprotective effects of *S. argentea* extract.

## 2. Materials and Methods

### 2.1. Extraction of S. argentea Using Solvents of Different Polarities

The roots and stems of *S. argentea* were ground into a fine powder and passed through a 40-mesh sieve. The resulting powder was soaked in 85% ethanol for 48 h to extract bioactive compounds. To enhance extraction efficiency, ultrasonic extraction was performed using an ultrasonic bath (KQ2200DE; Shumei Ultrasonic Instrument Co., Ltd., Kunshan, China) at a power of 100 W for 30 min per cycle. The ultrasonic amplitude was set to 100%. This process was repeated three times within a 48 h period. To minimize temperature-induced degradation, the samples were kept on ice water during sonication. Subsequently, the extract was concentrated using a rotary evaporator, yielding a 9.4% (EtOH) crude extract. This crude extract was further dissolved in distilled water and subjected to sequential extraction using petroleum ether, ethyl acetate, n-butanol, and water, resulting in specific fractions. The obtained fractions were designated as Petroleum Ether Extract (PE) (yield, 13%), Ethyl Acetate Extract (EAC) (yield, 8.3%), and n-Butanol Extract (NBA) (yield, 5.2%). The remaining extract was water-soluble (H2O). For further analysis, the ethanol and petroleum ether extracts were redissolved in dimethyl sulfoxide (DMSO), the ethyl acetate and n-butanol extracts were redissolved in methanol, and the aqueous extract was dissolved in distilled water to ensure complete solubility.

### 2.2. Analysis of Total Phenol and Flavonoid Contents

The total phenolic content of the extracts was determined using Folin–Ciocalteu reagent. The procedure involved mixing the extract with Folin–Ciocalteu reagent (200 μL) and allowing the mixture (20 μL) to stand for 5 min. Subsequently, 20% sodium carbonate (180 μL) was added. The mixture was then centrifuged at 12,000× *g* for 10 min to separate the supernatant. The absorbance was measured at 735 nm (OD735) using a microplate reader (Infinite 200Pro; Tecan, Männedorf, Switzerland), with gallic acid serving as the standard control. For the detection of total flavonoids, the Plant Flavonoids Content Assay Kit (Beijing Solarbio Science & Technology, Beijing, China) was utilized. The determination of total flavonoids is based on the formation of a red chelate complex between flavonoids and aluminum ions in an alkaline nitrite solution. Briefly, the extract was mixed with the provided reagents and incubated at 37 °C for 45 min. After centrifugation at 10,000× *g* for 10 min, the absorbance of the supernatant was measured at 470 nm against a rutin standard.

### 2.3. Analysis of ABTS and DPPH Scavenging Rates

The ABTS radical scavenging rate was determined using the Total Antioxidant Capacity Assay Kit with ABTS (Beyotime Biotechnology Co., Ltd., Shanghai, China), following the manufacturer’s instructions. The absorbance at 734 nm was measured using a microplate reader (Infinite 200Pro, Tecan). For the DPPH radical scavenging assay, DPPH was dissolved in anhydrous ethanol and mixed with different concentrations of the extract or bioactive compounds. The absorbance at 517 nm was measured using a microplate reader. Appropriate solvent blanks were used as controls to calculate the antioxidant capacity of *S. argentea* extracts and bioactive compounds.

### 2.4. Cell Viability Assessment

The viability of HaCaT cells was assessed using the MTT assay. HaCaT cells were cultured in Dulbecco’s Modified Eagle Medium (DMEM) supplemented with 10% fetal bovine serum (FBS) at 37 °C in a 5% CO_2_ incubator overnight. The cells were exposed to the varying concentrations of extracts and bioactive compounds in DMEM medium without FBS for 1 h and 12 h, after which the medium was aspirated. To each well, 90 μL of DMEM without FBS and 10 μL of a 5 mg/mL MTT solution were added, and the plates were incubated at 37 °C for 4 h. After incubation, the supernatant was removed, and 200 μL of DMSO was added to each well to dissolve the formazan crystals. The plates were then shaken for 10 min to ensure complete dissolution, and OD at 490 nm was measured using a microplate reader.

### 2.5. Cellular ROS Assay

To evaluate the impact of extracts and bioactive compounds on ROS production in UVB-irradiated HaCaT cells, the ROS Assay Kit with CM-H2DCFDA (Beyotime Biotechnology) was utilized. Initially, HaCaT cells were seeded into 96-well plates and incubated overnight at 37 °C in a 5% CO_2_. Afterward, the medium was aspirated, and DMEM without FBS was added, followed by the introduction of varying concentrations of extracts and bioactive compounds for 1 h. Subsequently, the cells were subjected to UVB irradiation using the Bio Sun system (Vilber Bio Imaging, France) at a dose of 225 mJ/cm^2^. A UVB dose of 225 mJ/cm^2^ was used for further experiments due to its induction of cellular damage and oxidative stress without excessive cell death. Following irradiation, the cells were further incubated for 1 h at 37 °C in a 5% CO_2_ incubator. The ROS Assay Kit was employed, with the DCFH-DA probe diluted to a final concentration of 10 μM in serum-free DMEM medium at a ratio of 1:1000. After removing the medium, the DCFH-DA solution was added to each well and incubated at 37 °C for 30 min. After incubation, the cells were washed three times with serum-free medium to eliminate the residual probes. The ROS levels were then quantified using a fluorescent microplate reader, with an excitation wavelength of 488 nm and an emission wavelength of 525 nm (Infinite 200Pro, Tecan). Moreover, a fluorescence microscope (DMi8; Leica Camera AG, Wetzlar, Germany) was utilized to visualize the cells and capture fluorescence images of the cells, indicating ROS generation.

### 2.6. UPLC-QTOF-MS Assay of the NBA Extract

The analysis of the n-butanol extract was performed using a Waters Acquity I-Class PLUS ultra-high-performance liquid tandem Waters Xevo G2-XS QTof high resolution mass spectrometer (Waters, Milford, MA, USA) entrusted to Biomarker Technologies Co., Ltd. (Beijing, China). The liquid chromatographic conditions were as follows: mobile phase A was a 0.1% formic acid solution, and mobile phase B was a 0.1% formic acid acetonitrile solution; the gradient elution was programmed as 2% mobile phase B from 0 to 0.25 min, increasing to 98% mobile phase B from 0.25 to 10 min, holding at 98% mobile phase B from 10 to 13.0 min, decreasing to 2% mobile phase B from 13.0 to 13.1 min, and maintaining 2% mobile phase B from 13.1 to 15 min. Mass spectra acquisition from both primary and secondary mass spectrometry data was conducted using Waters MassLynx v4.2 software, with the capillary voltage set at 2.0 kV for the positive-ion mode and −1.5 kV for the negative-ion mode, a cone voltage of 30 V, an ion source temperature of 150 °C, a desolvation gas temperature of 500 °C, a backflush gas flow rate of 50 L/h, and a desolventizing gas flow rate of 800 L/h. Compound identification was performed using Progenesis QI’s online METLIN database and a custom library from Biomarker Technologies Co., Ltd., with theoretical fragment ions considered. The mass error was controlled within 100 ppm. Prior to analysis, data were normalized using total ion current normalization.

### 2.7. Western Blot Analysis

HaCaT cells were treated with varying concentrations of bioactive compounds for 1 h. Afterward, the cells were subjected to UVB radiation at a dose of 225 mJ/cm^2^. After UVB exposure, the cells were cultivated at 37 °C with 5% CO_2_ for 1 h. The culture medium was then removed, and the cells were gently washed twice with PBS. Cell lysates were prepared using a lysis buffer containing 0.5% glycerol, 1% Triton X-100, 20 mM NaF, 150 mM NaCl, 2 mM Na₃VO₄, 50 mM Tris-HCl (pH 7.4), 0.1 mM bovine serum albumin, and 2 mM PMSF. The lysates were incubated on ice for 5 min, followed by sonication (Vibra-Cell, Sonics & Materials, Inc., Newtown, CT, USA) for cell disruption. The lysates were centrifuged at 13,000× *g* for 10 min, and the supernatant was collected for further analysis. Proteins in the supernatant were separated by SDS-PAGE and transferred to a membrane for Western blot analysis. Primary antibodies against p38, phosphorylated p38 (p-p38), JNK, phosphorylated JNK (p-JNK), and β-actin were diluted to a ratio of 1:1000 dilution. Antibodies for p38, p-p38, and JNK were obtained from ABClonal (Wuhan, China), while the p-JNK antibody was sourced from Cell Signaling Technology (Danvers, MA, USA). A peroxidase-conjugated rabbit IgG secondary antibody, diluted 1:5000, was utilized (Jackson ImmunoResearch Inc., West Grove, PA, USA). Protein detection was carried out using an enhanced chemiluminescence (ECL) chemiluminescence reagent (NcmECL Ultra; New Cell & Molecular Biotech Co., Ltd., Suzhou, China), and the corresponding images were captured with a ChemiDoc™ XRS+ System (Version 6.0) equipped with Image Lab™ Software (Version 6.1) (Bio-Rad, Hercules, CA, USA). The intensity of protein bands, reflecting expression levels, was quantified using ImageJ 1.53e software.

### 2.8. Analysis of JNK1 and p38 α Kinase Activity

To assess the impact of bioactive compounds on the activities of JNK1 and p38α kinase, the JNK1 Kinase Enzyme System, p38α Kinase Enzyme System, and ADP Glo™ Kinase Assay (Promega, Fitchburg, WI, USA) were employed. The experiment was conducted in white 384-well plates. The operation was carried out according to the instructions of the kit. The luminescence was detected using a fluorescent microplate reader (Infinite 200Pro, Tecan), with an integration time of 0.5–1 s. The production of ADP was monitored, and the luminescence signals were used to quantify the inhibitory effects of the bioactive compounds on kinase activity.

### 2.9. Cell Viability and Cytotoxicity Assay Using Calcein/PI

To assess the impact of bioactive compounds on HaCaT cell viability under UVB irradiation, the Calcein/PI Live/Dead Viability/Cytotoxicity Assay Kit (Beyotime Biotechnology) was utilized for fluorescence detection. HaCaT cells were treated with varying concentrations of bioactive compounds for 1 h. Afterward, the cells were subjected to UVB radiation at a dose of 225 mJ/cm^2^. Following UVB exposure, the cells were cultivated at 37 °C with 5% CO_2_ for 12 h. After incubation, the cells were stained using the Calcein/Propidium Iodide (PI) Kit. The medium was aspirated, and the cells were gently washed once with PBS. The Calcein/PI working solution was prepared according to the kit’s instructions, and 100 μL of this solution was added to each well for staining. The cells were then incubated at 37 °C in the dark for 30 min. The fluorescent staining of the cells was observed under a fluorescent microscope (DMI8, Leica).

### 2.10. Cell Apoptosis Assay Using Hoechst Staining

To assess the impact of bioactive compounds on HaCaT cell apoptosis under UVB irradiation, the Hoechst Staining Kit (Beyotime Biotechnology) was utilized for fluorescence staining and subsequent imaging. HaCaT cells were treated with varying concentrations of bioactive compounds for 1 h and then subjected to a UVB dose of 225 mJ/cm^2^. Subsequently, the cells were transferred to an incubator and maintained at 37 °C with 5% CO_2_ for 12 h. Afterward, the culture medium was removed, and the cells were treated according to the instructions of the kit. Apoptotic cells were analyzed through image analysis, and fluorescent images were captured under a fluorescent microscope (DMI8, Leica).

### 2.11. Cell Apoptosis Assay Using a Flow Cytometer

To assess the impact of bioactive compounds on HaCaT cell apoptosis under UVB irradiation, the Annexin V-FITC Apoptosis Detection Kit (Beyotime Biotechnology) was used for detection, and the results were analyzed by flow cytometry. After a 12 h incubation with bioactive compounds and UVB irradiation, 50,000 to 100,000 cells in suspension were collected and centrifuged at 1000× *g* for 5 min. The supernatant was then carefully removed, and the cell pellet was gently resuspended in 195 μL of Annexin V-FITC binding solution. Then, 5 μL of Annexin V-FITC and 10 μL of propidium iodide (PI) staining solution were introduced into the cell suspension. The mixture was incubated at room temperature in the dark for 10 to 20 min to allow for staining. Upon completion of the staining process, the ACEA NovoCyte flow cytometer (ACEA Biosciences, Inc., San Diego, CA, USA) was employed for detection and analysis.

### 2.12. Proteomic Analysis

The HaCaT cells, after a 12 h incubation with 200 μg/mL 3,4-dihydroxybenzoic acid and (+)-catechin and exposure to UVB irradiation, were collected for proteomic analysis. The conduction of this analysis was entrusted to MajorBio Co., Ltd. (Shanghai, China). Proteomic analysis was conducted utilizing Data-Independent Acquisition (DIA) and an Orbitrap Astral mass spectrometer (Thermo Fisher Scientific, Waltham, MA, USA). The mass spectrometry data underwent rigorous quality control. Data quality control and quantification were performed using proprietary software developed by MajorBio. Protein identification coverage and quantification accuracy were evaluated. Protein identification and functional annotation were performed using the UniProt database, while protein function analysis was integrated with insights from multiple databases, including evolutionary genealogy of genes: Non-supervised Orthologous Groups (eggNOG), Gene Ontology (GO), the Kyoto Encyclopedia of Genes and Genomes (KEGG), the non-redundant database (NR), the protein families database (Pfam), and a search tool for the retrieval of interacting genes/proteins (STRING). Quantitative results were evaluated using the Student’s *t*-test to identify differences between groups. The criteria for identifying proteins with significant differences were set as follows: absolute Log_2_ fold change (|Log_2_FC|) ≥ 1 and *p*-value < 0.05, as determined by R analysis. GO enrichment analysis was conducted using goatools to assess biological processes, molecular functions, and cellular components. KEGG pathway enrichment was analyzed using Python (Version 3.0) to identify significantly enriched signaling pathways.

### 2.13. Data Statistical Analysis

All experimental data are presented as means ± standard deviations. To compare the means across different groups, Duncan’s multiple range test was employed, with the significance level established at 95% confidence (*p* < 0.05). Statistical analyses were conducted utilizing IBM SPSS Statistics software (Version 27, SPSS Inc., Chicago, IL, USA).

## 3. Results

### 3.1. Antioxidant Capacities and Total Phenol and Flavonoid Contents of S. argentea Extracts

**Figure 1:**
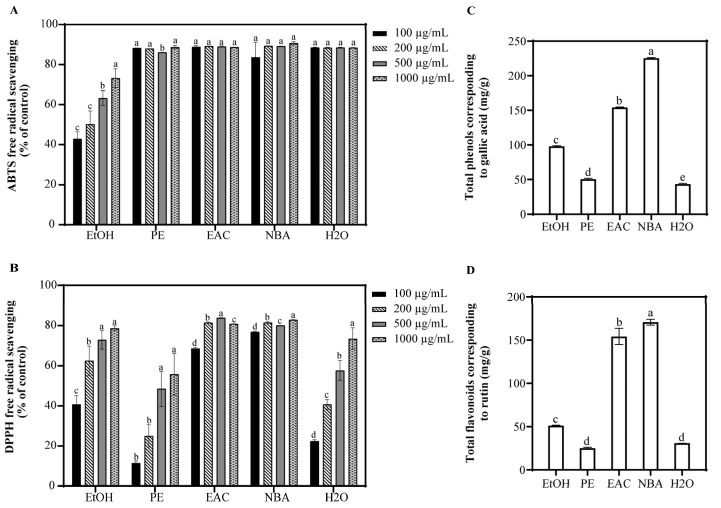
Antioxidant capacity and phytochemical composition of *S. argentea* extracts. (**A**) ABTS free radical scavenging of *S. argentea* extracts. (**B**) DPPH free radical scavenging of *S. argentea* extracts. (**C**) Total phenols of *S. argentea* extracts (expressed as gallic acid equivalents). (**D**) Total flavonoids of *S. argentea* extracts (expressed as rutin equivalents). EtOH, PE, EAC, NBA, and H2O indicate *S. argentea* extracts obtained using ethanol, petroleum ether, ethyl acetate, n-butanol, and distilled water, respectively. Different letters above the columns indicate statistically significant differences (*p* < 0.05). Results are presented as mean ± S.D. (n = 3).

To extract a broad spectrum of bioactive compounds, solvents with varying polarities were employed. Ethanol was used for general extraction, petroleum ether for non-polar compounds, ethyl acetate for moderately polar compounds, and n-butanol and water for highly polar compounds. The antioxidant capacities of EtOH, PE, EAC, NBA, and H2O extracts from *S. argentea* were assessed using ABTS and DPPH assays (Figure 1). At concentrations above 100 μg/mL, PE, EAC, NBA, and H2O showed over 83.6% ABTS radical scavenging, surpassing EtOH, which had a 73.2% rate at 1000 μg/mL. Among the extracts, NBA demonstrated strong DPPH radical scavenging, with rates exceeding 76.8% at concentrations above 100 μg/mL, followed by EAC, while PE showed the least effectiveness. Additionally, NBA had the highest levels of total phenols and flavonoids, suggesting its potent antioxidant properties and potential protective effects against oxidative stress and cell damage. Pearson correlation analysis revealed a significant positive correlation between total flavonoid content and total phenolic content (*p* < 0.001), confirming that flavonoids are a subset of polyphenols. Furthermore, DPPH radical scavenging activity was positively correlated with both total phenol and total flavonoid contents (*p* < 0.001), while no significant correlation was observed for ABTS. Consequently, NBA’s antioxidant efficacy under UVB-induced stress was further investigated through cell assays.

### 3.2. Effect of NBA on ROS Production and Survival Rate of UVB-Induced Cells

**Figure 2:**
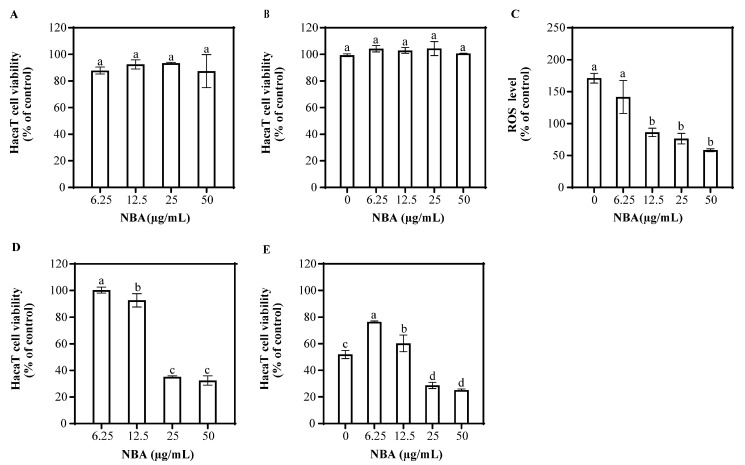
Effect of NBA on HaCaT cell viability and ROS generation. Cell viability of HaCaT cells treated with NBA in the (**A**) absence and (**B**) presence of UVB irradiation (225 mJ/cm^2^) after 1 h of incubation. (**C**) ROS levels in HaCaT cells treated with NBA after 1 h post-UVB treatment (225 mJ/cm^2^). The control was not exposed to UVB irradiation. Cell viability of HaCaT cells treated with NBA in the (**D**) absence and (**E**) presence of UVB irradiation (225 mJ/cm^2^) after 12 h of incubation. Different letters above the columns indicate statistically significant differences (*p* < 0.05). Results are presented as mean ± S.D. (n = 3).

NBA’s impact on HaCaT cell viability was assessed at various concentrations (Figure 2). At 50 μg/mL, NBA did not significantly affect cell survival. NBA treatment followed by UVB radiation also showed no significant difference in cell viability compared to the control after 1 h. NBA reduced ROS levels in a dose-dependent manner, with concentrations of 25 and 50 μg/mL reducing ROS to 76.5% and 58.3% of control levels, respectively.

After 12 h, NBA at concentrations of 6.25 and 12.5 μg/mL did not affect cell viability, but higher concentrations (25 and 50 μg/mL) decreased survival (Figure 2). NBA treatment at 6.25 μg/mL significantly improved cell survival after UVB radiation, increasing viability to 76.6%. In contrast, higher concentrations of NBA led to decreased survival, suggesting potential cytotoxicity at elevated doses.

### 3.3. Identification of NBA Components Using UPLC-QTOF-MS

To further analyze the chemical composition of NBA, UPLC-QTOF-MS was used to identify its components. The 15 compounds with the highest relative abundance are listed in Table 1. Beyond the notable presence of fatty acids and amino acids, the extract was also characterized by the presence of antioxidants, such as ellagic acid, quercetin, quercetin 3-arabinoside, sapondoside A, 3,4-dihydroxybenzoic acid, (+)-catechin, and procyanidin B2.

### 3.4. Antioxidant Capacities of Bioactive Compounds from NBA

**Figure 3:**
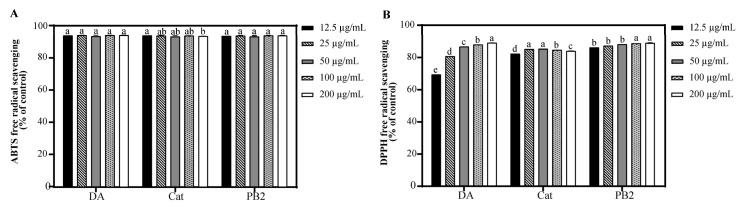
Antioxidant capacity of 3,4-dihydroxybenzoic acid, (+)-catechin, and procyanidin B2. (**A**) ABTS free radical scavenging of 3,4-dihydroxybenzoic acid, (+)-catechin, and procyanidin B2. (**B**) DPPH free radical scavenging of 3,4-dihydroxybenzoic acid, (+)-catechin, and procyanidin B2. The control was treated with H_2_O. DA, Cat, and PB2 indicate 3,4-dihydroxybenzoic acid, (+)-catechin, and procyanidin B2, respectively. Different letters above the columns indicate statistically significant differences (*p* < 0.05). Results are presented as mean ± S.D. (n = 3).

Quercetin, a principal component in the NBA, is widely recognized for its potent antioxidant and photoprotective properties, attributed to its exceptional free radical scavenging ability and protective effects against UVB-induced apoptosis. To evaluate whether other constituents within the NBA possessed similar antioxidant properties, the ABTS and DPPH radical scavenging activities of 3,4-dihydroxybenzoic acid, (+)-catechin, and procyanidin B2 were assessed (Figure 3). Our findings indicated that at a concentration of 12.5 µg/mL, all three compounds demonstrated excellent ABTS scavenging rates, exceeding 93.3%. Furthermore, in the DPPH scavenging analysis, (+)-catechin and procyanidin B2 showed outstanding antioxidant performance. At a concentration of 12.5 μg/mL, procyanidin B2 achieved a scavenging rate surpassing 86.2%, while (+)-catechin’s scavenging rate reached 82.4%. The antioxidant capacity of 3,4-dihydroxybenzoic acid ranked slightly lower but exhibited a dose-dependent increase, with its scavenging rate significantly rising from 69.5% at 12.5 μg/mL to 89.1% at 200 μg/mL. These results underscore the potential of these compounds as potent antioxidants within the NBA.

### 3.5. Effect of Bioactive Compounds on Cell Viability and ROS Generation of UVB-Induced Cells

**Figure 4:**
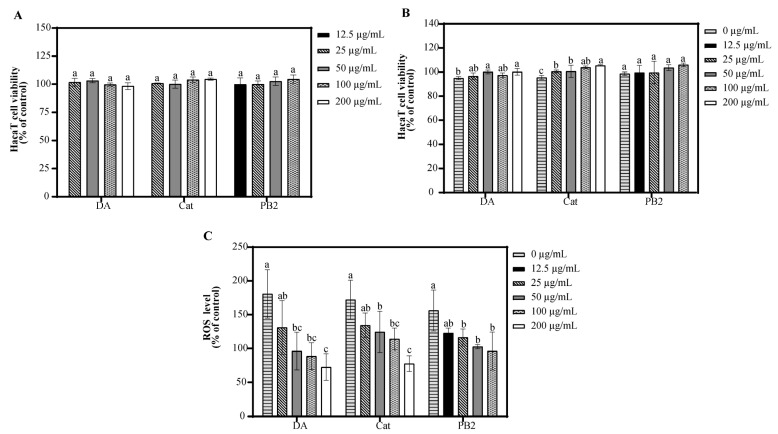
Effect of 3,4-dihydroxybenzoic acid, (+)-catechin, and procyanidin B2 on HaCaT cell viability and ROS generation. Cell viability of HaCaT cells treated with 3,4-dihydroxybenzoic acid, (+)-catechin, and procyanidin B2 in the (**A**) absence and (**B**) presence of UVB irradiation (225 mJ/cm^2^) after 1 h of incubation. (**C**) ROS levels in HaCaT cells treated with 3,4-dihydroxybenzoic acid, (+)-catechin, and procyanidin B2 after 1 h post-UVB treatment (225 mJ/cm^2^). Control groups were not exposed to UVB irradiation. Different letters above the columns indicate statistically significant differences (*p* < 0.05). Results are presented as mean ± S.D. (n = 3).

**Figure 5:**
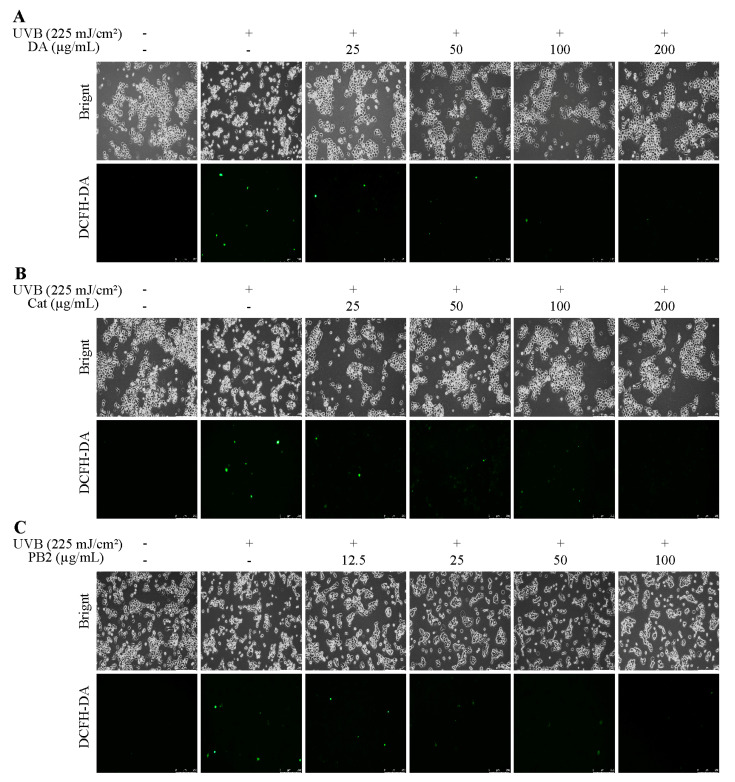
ROS assays of HaCaT cells treated with (**A**) 3,4-dihydroxybenzoic acid, (**B**) (+)-catechin, and (**C**) procyanidin B2 were assessed using a fluorescence microscope after 1 h post-UVB treatment (225 mJ/cm^2^).

In the MTT assay, 3,4-dihydroxybenzoic acid and (+)-catechin at 200 µg/mL showed over 95.3% cell survival, indicating no significant toxicity to HaCaT cells, while procyanidin B2 at the same concentration had a survival rate of 71.8%, suggesting cytotoxicity (Figure S1). Thus, 3,4-dihydroxybenzoic acid and (+)-catechin were determined at 25–200 µg/mL and procyanidin B2 at 12.5–100 µg/mL for further antioxidant studies. Following UVB irradiation and 1 h incubation, pre-treatment with these compounds resulted in cell survival rates over 94.9%, indicating no significant cytotoxicity (Figure 4). All three compounds showed the ability to inhibit ROS production in a dose-dependent manner, with 3,4-dihydroxybenzoic acid reducing ROS to 88.7% at 100 µg/mL, while procyanidin B2 and (+)-catechin had weaker effects, reducing ROS to 96.2% and 114.1%, respectively. Fluorescence microscopy using the 2′,7′-dichlorodihydrofluorescein diacetate (DCF-DA) probe confirmed that UVB radiation increased intracellular ROS levels. Treatment with the compounds resulted in a dose-dependent decrease in fluorescence intensity, further supporting their inhibitory effects on UVB-induced ROS generation (Figure 5).

### 3.6. Effect of Bioactive Compounds on the Expression and Activity of p38 and JNK

**Figure 6:**
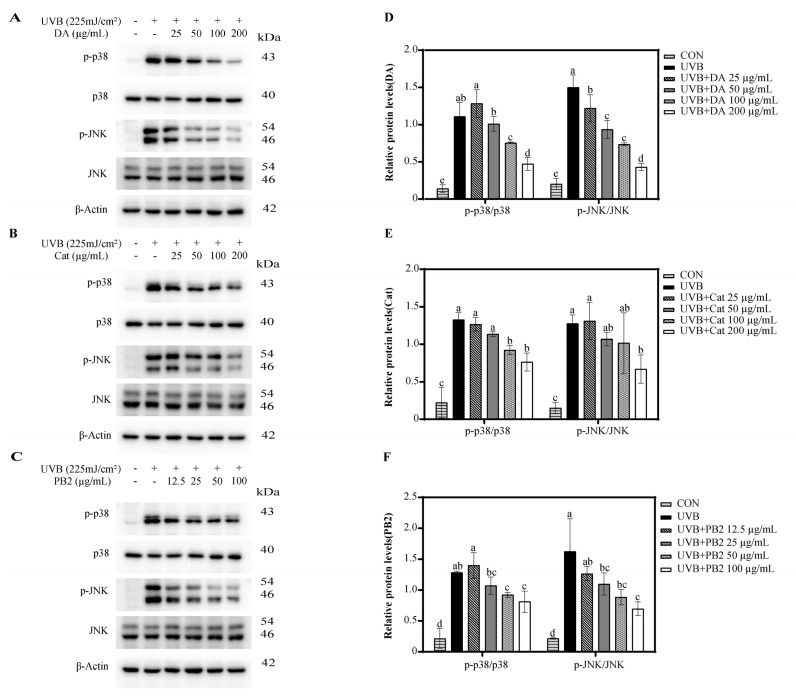
Western blot analysis of p-p38 and p-JNK expression in HaCaT cells treated with (**A**) 3,4-dihydroxybenzoic acid, (**B**) (+)-catechin, and (**C**) procyanidin B2 after 1 h post-UVB treatment (225 mJ/cm^2^). The relative intensities of proteins treated with (**D**) 3,4-dihydroxybenzoic acid, (**E**) (+)-catechin, and (**F**) procyanidin B2 were analyzed using ImageJ 1.53e software. Different letters above the columns indicate statistically significant differences (*p* < 0.05). Results are presented as mean ± S.D. (n = 3).

Western blot analysis showed that UVB radiation significantly upregulated the expression of phosphorylated p38 (p-p38) and phosphorylated JNK (p-JNK), thereby activating the p38 and JNK signaling pathways (Figure 6). 3,4-Dihydroxybenzoic acid, (+)-catechin, and procyanidin B2 demonstrated a dose-dependent inhibitory effect on the phosphorylation of p38 and JNK, with significant reductions compared to the UVB-only group. At a concentration of 100 μg/mL, 3,4-dihydroxybenzoic acid exhibited the most pronounced dephosphorylation effect, enhancing p38 dephosphorylation by 1.5-fold and JNK dephosphorylation by 2.0-fold compared to the UVB-only group. Similarly, at the same concentration, (+)-catechin and procyanidin B2 induced significant dephosphorylation of p38 by 1.4-fold. For JNK dephosphorylation, the respective increases were 1.3-fold and 1.6-fold. Thus, the dephosphorylation effect of 3,4-dihydroxybenzoic acid was superior to that of (+)-catechin and procyanidin B2.

The inhibitory effects of the compounds on p38α and JNK1 kinase activities were also assessed over a concentration range of 100 to 800 μg/mL. 3,4-Dihydroxybenzoic acid exhibited potent inhibitory activity against p38α kinase, achieving nearly complete inhibition at 400 and 800 μg/mL (Figure S2). In contrast, (+)-catechin and procyanidin B2 had weaker inhibitory effects, with maximum inhibition rates of 58.5% and 60.2% at 800 μg/mL, respectively. Regarding JNK1 kinase inhibition, 3,4-dihydroxybenzoic acid again showed potent activity, achieving 86.3% and 98.6% inhibition at 400 and 800 μg/mL, respectively. (+)-Catechin reached 62.8% inhibition at 800 μg/mL, while procyanidin B2 showed only 21.8% inhibition at the same concentration. The compounds’ effects were more pronounced at higher concentrations, suggesting they may modulate MAPK pathway activity indirectly, possibly through upstream proteins or by reducing oxidative stress.

### 3.7. Effect of Bioactive Compounds on Cellular Viability and Apoptosis

**Figure 7:**
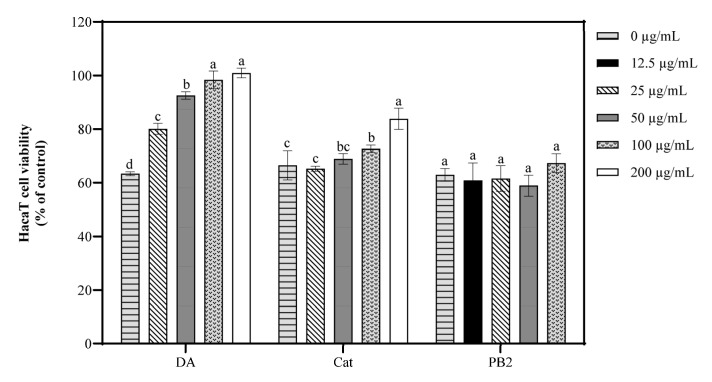
Viability of HaCaT cells treated with 3,4-dihydroxybenzoic acid, (+)-catechin, and procyanidin B2 was assessed after 12 h post-UVB treatment (225 mJ/cm^2^). Control groups were not exposed to UVB irradiation. DA, Cat, and PB2 indicate 3,4-dihydroxybenzoic acid, (+)-catechin, and procyanidin B2, respectively. Different letters above the columns indicate statistically significant differences (*p* < 0.05). Results are presented as mean ± S.D. (n = 3).

**Figure 8:**
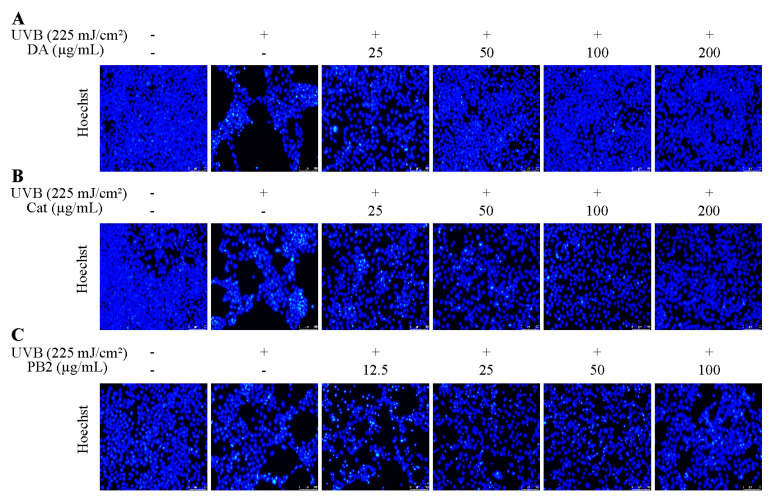
Hoechst staining of HaCaT cells treated with (**A**) 3,4-dihydroxybenzoic acid, (**B**) (+)-catechin, and (**C**) procyanidin B2 was assessed using fluorescence microscope after 12 h post-UVB treatment (225 mJ/cm^2^).

**Figure 9:**
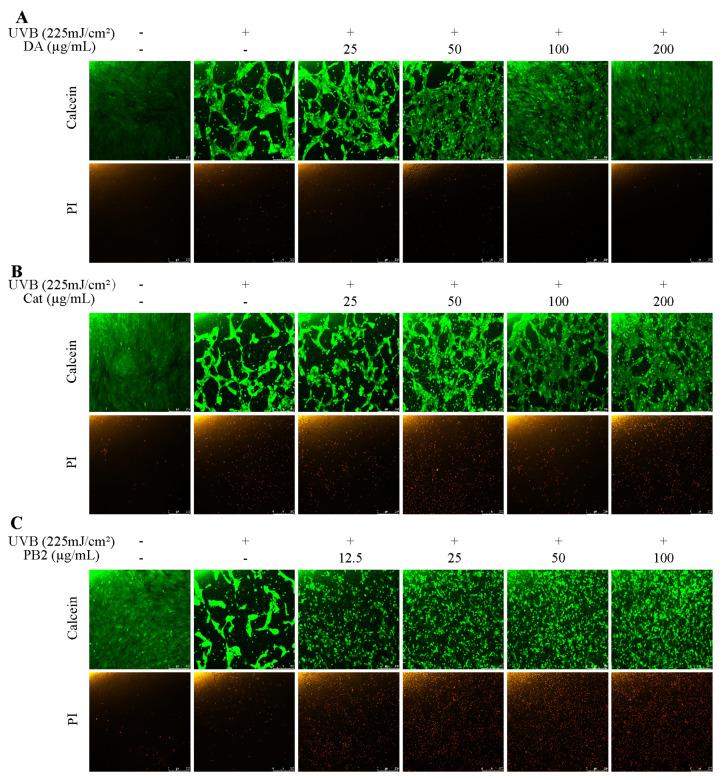
Calcein/PI staining of HaCaT cells treated with (**A**) 3,4-dihydroxybenzoic acid, (**B**) (+)-catechin, and (**C**) procyanidin B2 was assessed using fluorescence microscope after 12 h post-UVB treatment (225 mJ/cm^2^). Calcein and PI indicate viable and non-viable cells, respectively.

**Figure 10:**
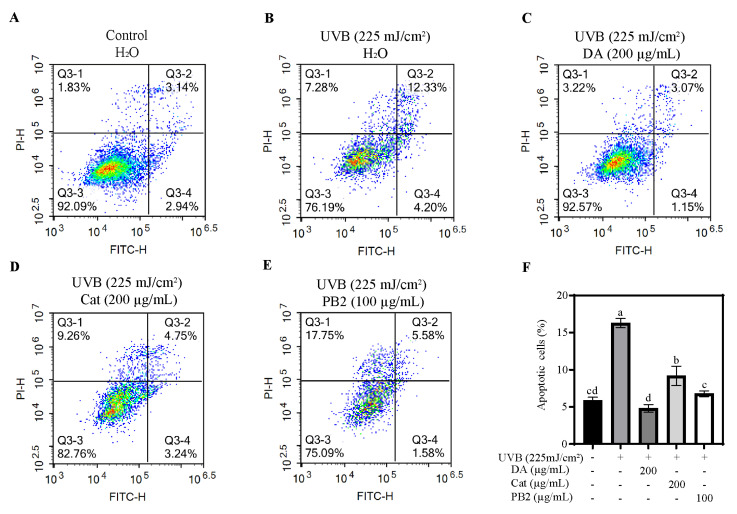
Flow cytometry analysis of HaCaT cells was assessed in the (**A**) absence and (**B**) presence of UVB irradiation (225 mJ/cm^2^) after 12 h of cultivation. Apoptosis of HaCaT cells treated with (**C**) 3,4-dihydroxybenzoic acid, (**D**) (+)-catechin, and (**E**) procyanidin B2 was assessed using flow cytometry after 12 h post-UVB treatment (225 mJ/cm^2^). Representative images are displayed. (**F**) The apoptotic cells were counted in quadrants Q2 and Q4. Different letters above the columns indicate statistically significant differences (*p* < 0.05). Results are presented as mean ± S.D. (n = 3).

The protective effects of 3,4-dihydroxybenzoic acid, (+)-catechin, and procyanidin B2 on HaCaT cells against UVB-induced stress were evaluated using various assays. In the MTT assay, 3,4-dihydroxybenzoic acid showed the most potent protective effect, enhancing cell survival to 80.1% at 25 μg/mL and fully restoring it to control levels at 200 μg/mL (Figure 7). (+)-Catechin was less effective, reaching 83.8% cell survival at 200 μg/mL, while procyanidin B2 showed no significant protection. In the Hoechst staining assay, 3,4-dihydroxybenzoic acid demonstrated dose-dependent protection against UVB-induced apoptosis, with nuclei nearly completely recovering at 100–200 μg/mL (Figure 8). (+)-Catechin provided less protection, and procyanidin B2 showed only marginal improvement at 100 μg/mL. In the Calcein/PI staining assay, 3,4-dihydroxybenzoic acid showed the strongest protective effect, with green fluorescence (viable cells) increasing and red fluorescence (non-viable cells) decreasing at high concentrations (Figure 9). (+)-Catechin was less effective, and procyanidin B2 showed high red fluorescence even at 100 μg/mL, indicating significant cell death. According to the analysis of flow cytometry with Annexin V-FITC staining, without UVB, cell survival was 92.4%, apoptosis 5.9%, and necrosis 1.7% (Figure 10). After UVB, survival dropped to 76.8%, apoptosis rose to 16.3%, and necrosis slightly increased. 3,4-Dihydroxybenzoic acid at 200 μg/mL restored survival to 92.2%, reduced apoptosis to 4.8%, and kept necrosis low. (+)-Catechin increased survival to 80.5% and reduced apoptosis to 9.2%, with a slight necrosis increase. Procyanidin B2 at 100 μg/mL had a survival rate of 73.9% and reduced apoptosis to 6.8%, but it increased necrosis to 19.4%. Overall, 3,4-dihydroxybenzoic acid was the most potent in protecting HaCaT cells against UVB-induced stress, while procyanidin B2 showed the least protection.

### 3.8. Proteomic Analysis of UVB-Induced Cells Treated with 3,4-Dihydroxybenzoic Acid and (+)-Catechin

Given the significant inhibition of UVB-induced cell apoptosis by 3,4-dihydroxybenzoic acid and (+)-catechin, proteomic analysis was employed to further elucidate their cellular effects. Proteomic analysis revealed that UVB irradiation led to the upregulation of 52 proteins and the downregulation of 273 (Figure S3). According to the analysis of heatmap clusters, treatment with 3,4-dihydroxybenzoic acid effectively modulated protein expression, restoring the proteomic profile closer to that of the control group, suggesting its role in reversing UVB-induced alterations. Functional annotation using GO enrichment analysis indicated that 3,4-dihydroxybenzoic acid enriched pathways related to RNA biosynthesis and DNA transcription regulation, implying its potential in alleviating UVB damage through DNA and RNA levels (Figure S4). (+)-Catechin was involved in pathways related to cell death regulation, stress response, and the extracellular matrix, indicating its role in cell death modulation and matrix remodeling. KEGG enrichment analysis showed that 3,4-dihydroxybenzoic acid was enriched in the p53 signaling and cell cycle pathways, suggesting its ability to prevent UVB-induced apoptosis by regulating cell cycle and DNA repair (Figure S5). (+)-Catechin was enriched in the phosphoinositide 3-kinase/protein kinase B (PI3K-Akt), wingless-related integration site (Wnt) signaling, and cell cycle pathways, implying its potential in mitigating oxidative stress by promoting cell survival and homeostasis. Key proteins associated with these pathways were significantly regulated by both compounds. 3,4-Dihydroxybenzoic acid upregulated proteins like CCND3, CCNB1, CCNB2, and CDK4 in cell cycle and p53 signaling (Table 2), while (+)-catechin upregulated proteins in cell repair and protection pathways, such as CDT1, YWHAZ, DDB2, and PKN3 (Table 3). This suggests that both compounds had specific mechanisms for protecting against UVB-induced stress, with 3,4-dihydroxybenzoic acid affecting cell cycle and DNA repair and (+)-catechin influencing cell survival and extracellular matrix remodeling. In addition, 3,4-dihydroxybenzoic significantly downregulated proteins such as c-Fos and Jun, which are known to promote apoptosis.

## 4. Discussion

UVB radiation significantly increases intracellular ROS levels, induces lipid peroxidation, disrupts mitochondrial membrane potential, and downregulates crucial antioxidant enzymes such as glutathione peroxidase 4 (GPX4) [19]. These detrimental effects ultimately lead to cell apoptosis, emphasizing the critical role of ROS generation and apoptotic signaling in UVB-induced skin damage [1,2,3]. Our study, through antioxidant assays and chemical composition analysis, revealed that the NBA demonstrated potent antioxidant properties. Notably, the NBA showed an ABTS and DPPH radical scavenging rate exceeding 76.8% at a concentration of 100 µg/mL, outperforming other extracts (Figure 1). The NBA extract was particularly rich in total phenols (225.3 mg/g) and total flavonoids (170.7 mg/g), as confirmed by UPLC-QTOF-MS composition analysis (Table 1). The robust free radical scavenging capacity of NBA was likely due to these active components.

Previous studies showed that total phenols and flavonoids from plant extracts, as key antioxidant constituents, can mitigate oxidative stress by removing free radicals and activating antioxidant enzyme systems, including superoxide dismutase (SOD), catalase (CAT), and glutathione peroxidase (GPx) [20]. Furthermore, they can enhance cellular antioxidant defenses by activating the Nrf2/ARE signaling pathway, thereby alleviating UVB-induced oxidative stress and significantly improving cell survival rates [12,13,21]. NBA extract also exhibited significant UV absorption in the UVB spectrum (280–315 nm), with a secondary absorption peak (Figure S6), suggesting the presence of compounds capable of absorbing UV radiation. Phenolic and flavonoid compounds, with their aromatic rings and hydroxyl groups, are effective at absorbing ultraviolet energy, reducing the direct cellular damage caused by UVB radiation [22]. NBA contained compounds such as ellagic acid, quercetin, quercetin 3-arabinolide, 3,4-dihydroxybenzoic acid, procyanidin B2, (+)-catechin, and aloin, which possessed UV-absorbing properties [23,24,25,26]. Quercetin, a major component of NBA, has been shown to significantly reduce ROS generation, protect mitochondrial function, inhibit the activation of apoptotic signaling pathways, and alleviate UVB-induced cell damage in oxidative stress models [27,28]. It may also defend against UV-B-induced oxidative stress through Nrf2-dependent pathways [29]. Consequently, NBA extract effectively reduced ROS and mitigated UVB-induced damage, as shown in Figure 2. According to these findings, we further investigated whether 3,4-dihydroxybenzoic acid, (+)-catechin, and procyanidin B2 share similar photoprotective effects on HaCaT cells.

3,4-Dihydroxybenzoic acid, (+)-catechin, and procyanidin B2 have been demonstrated to reduce ROS levels with a dose-dependent effect, as validated by fluorescence analysis (Figure 4 and Figure 5). This finding aligns with similar evidence from previous studies. In L929 cells, 3,4-dihydroxybenzoic acid and its derivatives were found to significantly suppress ROS generation, suggesting that its antioxidant mechanism involves a direct decrease in free radicals, the inhibition of NADPH oxidase activity, and the restoration of cellular redox balance [30]. Wu et al. found that exposure to UVB radiation led to a significant increase in H_2_O_2_ levels in HaCaT cells, with an elevation of 1.3 to 1.5 times compared to the control. However, high concentrations of (+)-catechin (100 μM) were revealed to substantially reduce H_2_O_2_ levels, bringing them closer to those of the control group [31]. Procyanidin B2, on the other hand, has been shown to effectively mitigate heat-stress-induced ROS accumulation. It significantly improved the activity of antioxidant enzymes such as catalase and superoxide dismutase by activating the Nrf2 signaling pathway, thus diminishing oxidative damage [32].

In the Western blot analysis, our results revealed that UVB radiation markedly enhanced the phosphorylation levels of p38 and JNK, in agreement with previous studies [13,33]. Further investigation indicated that 3,4-dihydroxybenzoic acid, (+)-catechin, and procyanidin B2 significantly suppressed the phosphorylation of p38 and JNK under high-concentration conditions, highlighting their robust regulatory influence on the MAPK pathway (Figure 6). However, when examining the direct inhibitory effects of these compounds on p38α and JNK1 kinase activities, significant inhibition was observed only at high concentrations (Figure S2). The half-maximal inhibitory concentration (IC50) values for p38α were 266.4 μg/mL for 3,4-dihydroxybenzoic acid, 716.5 μg/mL for (+)-catechin, and 595.5 μg/mL for procyanidin B2. For JNK1 inhibition, the IC50 values were 224.8 μg/mL for 3,4-dihydroxybenzoic acid and 510.6 μg/mL for (+)-catechin. At a concentration of 800 μg/mL, procyanidin B2 demonstrated only a 21.8% inhibition rate against JNK1. These findings suggest that the modulation of the MAPK signaling pathway by these compounds may primarily involve indirect mechanisms, rather than direct suppression of p38 and JNK activity.

Previously, UVB exposure has not been commonly employed to study the inhibitory effects of antioxidants on HaCaT cell apoptosis. In this study, we utilized a comprehensive approach, including the MTT assay, Hoechst staining, Calcein/PI fluorescence microscopy, and flow cytometry, to assess the protective efficacy of 3,4-dihydroxybenzoic acid and (+)-catechin against UVB-induced apoptosis in HaCaT cells. At a concentration of 200 μg/mL 3,4-dihydroxybenzoic acid, the cell survival rate was nearly restored to levels observed in the control. Moreover, 3,4-dihydroxybenzoic acid effectively protected HaCaT cells from UVB radiation damage by reducing apoptosis and maintaining membrane integrity. (+)-Catechin demonstrated a similar protective effect, while procyanidin B2 did not exhibit a preventative effect against UVB-induced apoptosis. Previous studies have shown that 3,4-dihydroxybenzoic acid, isolated from *Cladophora wrightiana* Harvey, significantly mitigated the activity loss of HaCaT cells under low-dose UVB radiation (30 mJ/cm^2^) and demonstrated promising anti-apoptosis effects [34]. Mittraphab et al. also found that 3,4-dihydroxybenzoic acid protected HaCaT cells from UVB damage by reducing ROS accumulation and inhibiting the expression of inflammatory markers such as COX-2 and inducible nitric oxide synthase (iNOS) under similar UVB doses (30 mJ/cm^2^) [35]. On the other hand, (+)-catechin has been reported to significantly inhibit H₂O₂-induced fibroblast apoptosis by suppressing the phosphorylation of JNK and p38 signaling pathways triggered by oxidative stress and by decreasing caspase-3 activation [36]. Similarly, (+)-catechins significantly increased the survival rate of human keratinocytes at lower UVB doses (50 mJ/cm^2^) by inhibiting JNK phosphorylation and scavenging ROS [31].

Although Procyanidin B2 did not effectively reduce cell apoptosis under high-dose UVB radiation in this study, it has been shown to significantly reduce cell apoptosis in ARPE-19 cells induced by blue light. This protective effect is attributed to the inhibition of the mitochondrial apoptosis pathway (e.g., increasing the B-cell lymphoma 2 (BCL-2)/Bcl-2-associated X protein (BAX) ratio and reducing caspase activation), alleviation of endoplasmic reticulum stress, and inhibition of cell apoptosis through the activation of the unfolded protein response (UPR) [37]. However, in our study, flow cytometry analysis revealed that while procyanidin B2 reduced the apoptosis rate, it was accompanied by a significant increase in the necrosis rate (19.4%), suggesting potential phototoxicity (Figure 10). Lai et al.’s study found that the combined treatment of glycolic acid and UVB significantly intensified the apoptosis of HaCaT cells. The synergistic phototoxic effect triggers cell apoptosis through multiple pathways, including mitochondria- and endoplasmic reticulum-dependent pathways, as well as both caspase-dependent and caspase-independent pathways [38]. Through Calcein/PI dual staining observation, it was found that although procyanidin B2 increased the proportion of surviving cells (green fluorescence) at a concentration of 100 μg/mL, the proportion of dead cells (red fluorescence) remained high (Figure 9). Additionally, the procyanidin B2 treatment exhibited significant changes in cell morphology, including obvious cell shrinkage and deformation, further indicating that procyanidin B2 may exacerbate cell damage through photochemical reactions.

Proteomic analysis was further employed to elucidate the molecular mechanisms underlying the cell-protective effects of 3,4-dihydroxybenzoic acid and (+)-catechin. KEGG enrichment analysis revealed significant enrichment of 3,4-dihydroxybenzoic acid in the “cell cycle”, “p53 signaling pathway”, and “pathways in cancer” pathways (Table 2). This suggests that 3,4-dihydroxybenzoic acid had a notable potential to counteract UVB damage by modulating the cell cycle and bolstering DNA repair functions [25]. Furthermore, 3,4-dihydroxybenzoic acid effectively downregulated the expression of c-Fos and Jun, thereby inhibiting the overactivity of the AP-1 complex and subsequently regulating downstream molecules like the p53-p21 pathway. This regulation provided protection against UVB-induced oxidative stress, cell cycle arrest, and apoptotic responses [6,7]. Chen et al. have shown that the p38 MAPK inhibitor SB202190 effectively reduces the activity of c-Fos and AP-1, thereby alleviating UVB-induced oxidative stress [6]. A similar mechanism was confirmed in the study by Park et al. (2016), where 3,4-dihydroxybenzoic acid was found to significantly downregulate the expression of nuclear factor of activated T-cells, cytoplasmic 1 (NFATc1) [39]. This was achieved by inhibiting JNK phosphorylation, decreasing the stability of c-Fos protein, and reducing AP-1 activity. Consequently, it mitigated the inflammatory bone loss caused by receptor activator of NF-κB ligand-induced osteoclast differentiation.

KEGG enrichment analysis revealed that (+)-catechin was enriched in the “PI3K-Akt signaling pathway,” “Wnt signaling pathway,” and “cell cycle” pathway (Table 3). This suggests that (+)-catechin mitigated UVB-induced oxidative stress and cell damage by synergistically regulating multiple signaling pathways. Specifically, regarding cell cycle regulation, (+)-catechin upregulated the expression of CDT1 and YWHAZ, which accelerated cell cycle progression and promoted damage repair [40,41]. In the p53 signaling pathway, the upregulation of DDB2 and RCHY1 significantly enhanced DNA repair capabilities [42,43]. Furthermore, the upregulation of YWHAZ and PKN3 within the PI3K-Akt signaling pathway demonstrated potent anti-apoptotic and cell survival maintenance effects [41,44,45,46]. Additionally, the upregulation of BTRC and TBL1X within the Wnt signaling pathway may further alleviate UVB damage by regulating cell proliferation and differentiation [47,48]. Previous studies have demonstrated that (+)-catechins activate the PI3K-Akt signaling pathway and upregulate the expression of proteins such as Pdx-1 and MafA, thereby promoting cell proliferation and survival. Concurrently, they effectively inhibit cell apoptosis by regulating downstream glycogen synthase kinase-3 beta (GSK3β) and tribbles-3 (TRB3) [49]. Moreover, (+)-catechins can significantly increase the expression of antioxidant enzymes, such as heme oxygenase-1 (HO-1) and NAD(P)H quinone dehydrogenase 1 (NQO1), by activating the PI3K/Akt signaling pathway and its downstream Nrf2/HO-1. This effectively clears ROS and alleviates oxidative stress, while also significantly reducing the inflammatory response by inhibiting the activation of NF-κB [50].

This study demonstrated that 3,4-dihydroxybenzoic acid and (+)-catechin, isolated from the n-butanol extract of *S. argentea*, exhibited significant photoprotective effects against UVB-induced damage in HaCaT cells. Both compounds effectively reduced ROS levels, inhibited p38 and JNK phosphorylation, and protected cells from apoptosis. However, the study primarily focused on the in vitro effects of these isolated compounds, which may not fully reflect the complex interactions within the NBA extract of *S. argentea*. Further investigation into the synergistic or antagonistic effects among multiple compounds in the extract is essential for a more comprehensive understanding of its biological activity. Despite these limitations, this study provides valuable insights into the photoprotective mechanisms of specific compounds from *S. argentea*, laying the foundation for future research on developing novel photoprotective agents from natural sources.

## 5. Conclusions

This study explored the photoprotective effects of the n-butanol extract of *S. argentea* (NBA) and its bioactive compounds—3,4-dihydroxybenzoic acid, (+)-catechin, and procyanidin B2—against UVB-induced oxidative stress. Our research has determined that NBA extract is rich in total phenols (225.3 mg/g) and flavonoids (170.7 mg/g), which contribute to its significant free radical scavenging ability and antioxidant effects. This was the first study to elucidate its antioxidant and anti-apoptotic mechanisms through the synergistic regulation of multiple signaling pathways by proteomic analysis. Specifically, 3,4-dihydroxybenzoic acid bolstered DNA repair ability and markedly reduced ROS generation by 92.1% at 100 μg/mL and improved cell survival to 100.9% at 200 μg/mL. These effects were mediated by modulating the p53 signaling pathway and Fos/Jun expression. (+)-Catechin stimulated cell proliferation and survival by activating the PI3K-Akt and Wnt pathways, resulting in an 83.8% increase in cell viability at a concentration of 200 μg/mL. While procyanidin B2 exhibited antioxidant properties, it also demonstrated potential phototoxicity. At 100 μg/mL, procyanidin B2 did not significantly improve cell survival and instead increased necrosis to 19.4%, emphasizing the crucial need for safety considerations in the development of antioxidant agents. This study not only confirmed the photoprotective potential of NBA and its active ingredients against UVB damage but also uncovered the molecular mechanisms of their synergistic regulation across multiple signaling pathways. Future research should investigate the long-term protective effects and safety of these compounds in in vivo models to facilitate practical applications.

## Figures and Tables

**Table 1:** UPLC-QTOF-MS analysis of the n-butanol extract of *S. argentea*.

Ion Mode	No.	Compounds	Retention Time (min)	*m*/*z*	Relative Abundance
Negative	1	PG(16:0/0:0) [U]	9.074	483.272	1.779 × 10^6^ ± 1.401 × 10^5^
	2	3-O-Mycarosylerythronolide B	2.250	1151.765	7.988 × 10^5^ ± 3.729 × 10^5^
	3	Thr Asp Phe Glu	3.496	509.220	6.163 × 10^5^ ± 4.755 × 10^4^
	4	Ellagic acid	3.340	300.999	5.059 × 10^5^ ± 2.010 × 10^4^
	5	Quercetin 3-arabinoside	3.560	433.078	3.158 × 10^5^ ± 1.658 × 10^4^
	6	Nodularin	8.732	823.469	3.263 × 10^5^ ± 3.273 × 10^4^
	7	Hieracin	4.522	301.036	1.894 × 10^5^ ± 1.438 × 10^4^
	8	3,3-Dimethylglutaric acid	2.477	205.072	1.635 × 10^5^ ± 5.904 × 10^3^
	9	Procyanidin B2	2.670	577.135	1.183 × 10^5^ ± 1.417 × 10^4^
	10	Leukotriene F4	8.925	567.285	9.387 × 10^4^ ± 5.607 × 10^3^
	11	Aloin A	3.852	439.107	8.512 × 10^4^ ± 1.232 × 10^4^
	12	Sapindoside A	8.497	809.455	8.456 × 10^4^ ± 6.602 × 10^3^
	13	TyrMe-Met-OH	2.157	447.115	7.200 × 10^4^ ± 9.69 × 10^2^
	14	(+)-Catechin	2.855	289.072	5.214 × 10^4^ ± 3.94 × 10^2^
	15	3,4-Dihydroxybenzoic acid	2.228	153.019	5.750 × 10^4^ ± 5.411 × 10^3^
Positive	1	PC(16:0/20:4(5E,8E,11E,14E))	10.007	782.569	2.897 × 10^6^ ± 2.466 × 10^5^
	2	Lucidal	5.825	437.342	1.032 × 10^6^ ± 3.346 × 10^4^
	3	Harderoporphyrin	10.164	609.271	1.033 × 10^6^ ± 1.541 × 10^4^
	4	1-Linoleoylglycerophosphocholine	7.621	520.340	8.125 × 10^5^ ± 6.997 × 10^4^
	5	LysoPC(18:3(6Z,9Z,12Z))	7.129	518.325	7.264 × 10^5^ ± 5.692 × 10^4^
	6	Docosa-4,7,10,13,16-pentaenoyl carnitine	8.447	474.379	7.351 × 10^5^ ± 3.464 × 10^4^
	7	PG(16:1(9Z)/0:0)	8.490	447.251	6.402 × 10^5^ ± 1.171 × 10^4^
	8	Ricinoleic acid methyl ester	9.159	313.274	4.714 × 10^5^ ± 1.046 × 10^4^
	9	PC(0:0/18:0)	8.967	524.372	4.662 × 10^5^ ± 1.622 × 10^4^
	10	Leukotriene C4	10.022	625.267	4.749 × 10^5^ ± 7.798 × 10^3^
	11	Thr Trp Met Arg	10.598	593.277	3.980 × 10^5^ ± 4.239 × 10^3^
	12	Dehydro(11,12)ursolic acid lactone	6.125	437.342	3.726 × 10^5^ ± 2.402 × 10^3^
	13	Quercetin	3.660	303.051	3.505 × 10^5^ ± 6.453 × 10^3^
	14	Phaeophorbide B	9.537	607.256	3.154 × 10^5^ ± 1.193 × 10^4^
	15	MG(0:0/16:1(9Z)/0:0)	8.511	311.259	3.153 × 10^5^ ± 1.042 × 10^4^

**Table 2:** KEGG enrichment analysis of differentially expressed proteins in HaCaT cells treated with and without 3,4-dihydroxybenzoic acid after 12 h post-UVB treatment (225 mJ/cm^2^).

KEGG Description	Gene	Protein Name	FC	Log_2_FC	*p*-Value
Cell cycle	CDT1	DNA replication factor Cdt1	32.00	5.00	1.02 × 10^−7^
	CCND3	G1/S-specific cyclin-D3	32.00	5.00	5.38 × 10^−6^
	CCNB1	G2/mitotic-specific cyclin-B1	2.40	1.24	5.74 × 10^−5^
	-	Cell division control protein	32.00	5.00	1.84 × 10^−10^
	PTTG1	Pituitary tumor-transforming protein 1	3.61	1.85	2.78 × 10^−4^
	CDK4	Cyclin-dependent kinase 4	2.30	1.20	6.82 × 10^−5^
	CCNB2	G2/mitotic-specific cyclin-B2	2.14	1.10	1.43 × 10^−4^
	WEE1	Wee1-like protein kinase	3.48	1.80	4.24 × 10^−5^
	-	Aurora kinase	2.11	1.08	1.19 × 10^−4^
	AURKB	Aurora kinase B	2.05	1.04	1.64 × 10^−4^
	ESCO2	N-acetyltransferase ESCO2	3.17	1.67	4.97 × 10^−5^
	MCM4	DNA replication licensing factor MCM4	32.00	5.00	2.26 × 10^−6^
	ORC6	Origin recognition complex subunit 6	2.08	1.06	1.23 × 10^−3^
	CCNA2	Cyclin-A2	2.75	1.46	2.15 × 10^−6^
	CDC20	Cell division cycle protein 20 homolog	2.23	1.16	2.29 × 10^−6^
p53 signaling pathway	RCHY1	RING finger and CHY zinc finger domain-containing protein 1	32.00	5.00	1.01 × 10^−7^
	CCND3	G1/S-specific cyclin-D3	32.00	5.00	5.38 × 10^−6^
	CCNB1	G2/mitotic-specific cyclin-B1	2.36	1.24	5.74 × 10^−5^
	CDK4	Cyclin-dependent kinase 4	2.30	1.20	6.82 × 10^−5^
	CCNB2	G2/mitotic-specific cyclin-B2	2.14	1.10	1.43 × 10^−4^
	DDB2	DNA damage-binding protein 2	6.12	2.61	3.10 × 10^−4^
	ZNF385A	Zinc finger protein 385A	3.20	1.68	8.34 × 10^−4^
Pathways in cancer	CCND3	G1/S-specific cyclin-D3	32.00	5.00	5.38 × 10^−6^
	CDK4	Cyclin-dependent kinase 4	2.30	1.20	6.82 × 10^−5^
	GSTM4	Glutathione S-transferase Mu 4	2.23	1.16	1.70 × 10^−2^
	DDB2	DNA damage-binding protein 2	6.12	2.61	3.10 × 10^−4^
	STAT3	Signal transducer and activator of transcription	2.58	1.37	1.09 × 10^−6^
	STAT3	Signal transducer and activator of transcription	3.72	1.90	7.51 × 10^−3^
	LAMB3	Laminin subunit beta-3	2.02	1.01	3.81 × 10^−6^
	FOS	Protein c-Fos	0.17	−2.59	6.53 × 10^−4^
	NOTCH2	Neurogenic locus notch homolog protein 2	2.16	1.11	5.25 × 10^−3^
	-	JUN	0.45	−1.15	3.00 × 10^−4^
	CCNA2	Cyclin-A2	2.75	1.46	2.15 × 10^−6^
	LAMA5	Laminin subunit alpha-5	2.04	1.03	1.15 × 10^−4^
	GNA12	G protein subunit alpha 12	32.00	5.00	5.94 × 10^−7^

**Table 3:** KEGG enrichment analysis of differentially expressed proteins in HaCaT cells treated with and without (+)-catechin after 12 h post-UVB treatment (225 mJ/cm^2^).

KEGG Description	Gene	Protein Name	FC	Log_2_FC	*p*-Value
Cell cycle	CDT1	DNA replication factor Cdt1	32.00	5.00	1.59 × 10^−8^
	YWHAZ	Tyrosine 3-monooxygenase	2.03	1.02	1.58 × 10^−2^
	-	Cell division control protein	32.00	5.00	9.33 × 10^−7^
p53 signaling pathway	RCHY1	RING finger and CHY zinc finger domain-containing protein 1	32.00	5.00	4.52 × 10^−8^
	DDB2	DNA damage-binding protein 2	2.02	1.01	1.22 × 10^−2^
	-	E3 ubiquitin-protein ligase	2.43	1.28	3.11 × 10^−2^
PI3K-Akt signaling pathway	YWHAZ	Tyrosine 3-monooxygenase	2.03	1.02	1.58 × 10^−2^
	COL9A3	Collagen alpha-3(IX) chain	2.09	1.06	4.77 × 10^−3^
	PKN3	Serine/threonine-protein kinase N3	32.00	5.00	1.19 × 10^−3^
Pathways in cancer	DDB2	DNA damage-binding protein 2	2.02	1.01	1.22 × 10^−2^
	GNA12	G protein subunit alpha 12	32.00	5.00	8.86 × 10^−9^
Wnt signaling pathway	BTRC	Beta-transducin repeat containing isoform 3	32.00	5.00	2.58 × 10^−4^
	TBL1X	F-box-like/WD repeat-containing protein TBL1X	32.00	5.00	6.95 × 10^−8^
	-	E3 ubiquitin-protein ligase	32.00	5.00	5.85 × 10^−7^

## Data Availability

The raw data supporting the conclusions of this article will be made available by the authors on request.

## Supplementary Materials

The following supporting information can be downloaded at: https://www.mdpi.com/article/10.3390/antiox14020241/s1, Figure S1: Viability of HaCaT cells treated with different concentrations of 3,4-dihydroxybenzoic acid, (+)-catechin, and procyanidin B2 in the absence of UVB treatment after 12 h of cultivation; Figure S2: The inhibition rate of (A) p38α and (B) JNK1 kinase activities following treatment with different concentrations of 3,4-dihydroxybenzoic acid, (+)-catechin, and procyanidin B2; Figure S3: The statistics and heatmap clusters of differentially expressed proteins treated with 3,4-dihydroxybenzoic acid and (+)-catechin; Figure S4: GO enrichment analysis of differentially expressed proteins treated with 3,4-dihydroxybenzoic acid and (+)-catechin after 12 h post-UVB treatment (225 mJ/cm^2^); Figure S5: KEGG enrichment analysis of differentially expressed proteins treated with 3,4-dihydroxybenzoic acid and (+)-catechin after 12 h post-UVB treatment (225 mJ/cm^2^); Figure S6: Analysis of the absorption wavelengths for (A) NBA, (B) 3,4-dihydroxybenzoic acid, (C) (+)-catechin, and (D) procyanidin B2, ranging from 220 nm to 400 nm.

## Abbreviations

The following abbreviations are used in this manuscript:

NBA	n-Butanol Extract of Schima argentea
HaCaT	Human Adult Keratinocyte Cell Line
MAPK	Mitogen-Activated Protein Kinase
UPLC-QTOF-MS	Ultra-High-Performance Liquid Chromatography–Quadrupole Time-of-Flight Mass Spectrometry
MTT	3-(4,5-Dimethylthiazol-2-yl)-2,5-diphenyltetrazolium bromide
UVB	Ultraviolet B
JNK	c-Jun N-terminal kinase
c-Fos	Cellular Oncogene fos
PI3K-Akt	Phosphoinositide 3-Kinase/Protein Kinase B
Wnt	Wingless-Related Integration Site
ROS	Reactive Oxygen Species
ABTS	2,2′-Azino-bis(3-ethylbenzothiazoline-6-sulfonic Acid)
DPPH	2,2-Diphenyl-1-picrylhydrazyl
DMSO	Dimethyl Sulfoxide
Annexin V-FITC	Annexin V conjugated with Fluorescein Isothiocyanate
GO	Gene Ontology
KEGG	Kyoto Encyclopedia of Genes and Genomes
IC50	Half-Maximal Inhibitory Concentration
