# Supplementary material for: Antioxidant and Photoprotective Activities of 3,4-Dihydroxybenzoic Acid and (+)-Catechin, Identified from Schima argentea Extract, in UVB-Irradiated HaCaT Cells
Source: Antioxidants (Basel). 2025 Feb 19;14(2):241. doi: 10.3390/antiox14020241 (PMC11852075; doi:10.3390/antiox14020241)
Supplement: Supplementary file 1 [file antioxidants-14-00241-s001.zip › antioxidants-3448701-supplementary.pdf]

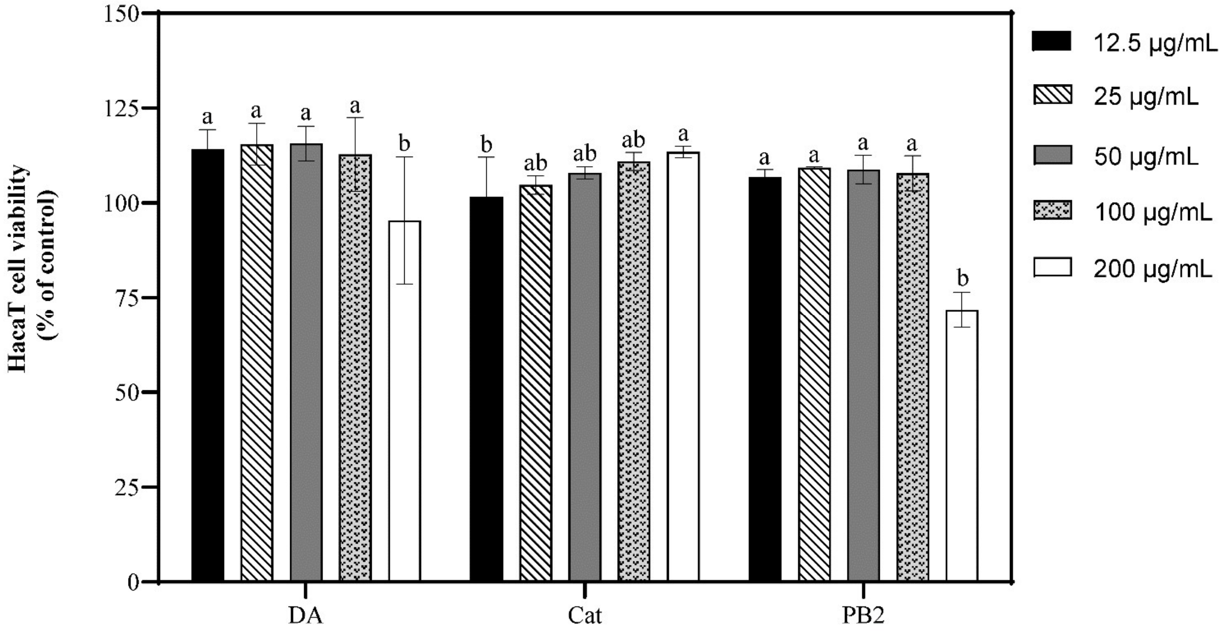

Figure S1. HaCaT cell viability treated with different concentrations of 3,4-dihydroxybenzoic acid, (+)-catechin, and procyanidin B2 in the absence of UVB treatment after 12 h of cultivation. DA, Cat, and PB2 indicate 3,4-dihydroxybenzoic acid, (+)-catechin, and procyanidin B2, respectively. Different letters above the columns indicate statistically significant differences ($p < 0.05$). Results are presented as mean ± S.D. ($n = 3$).

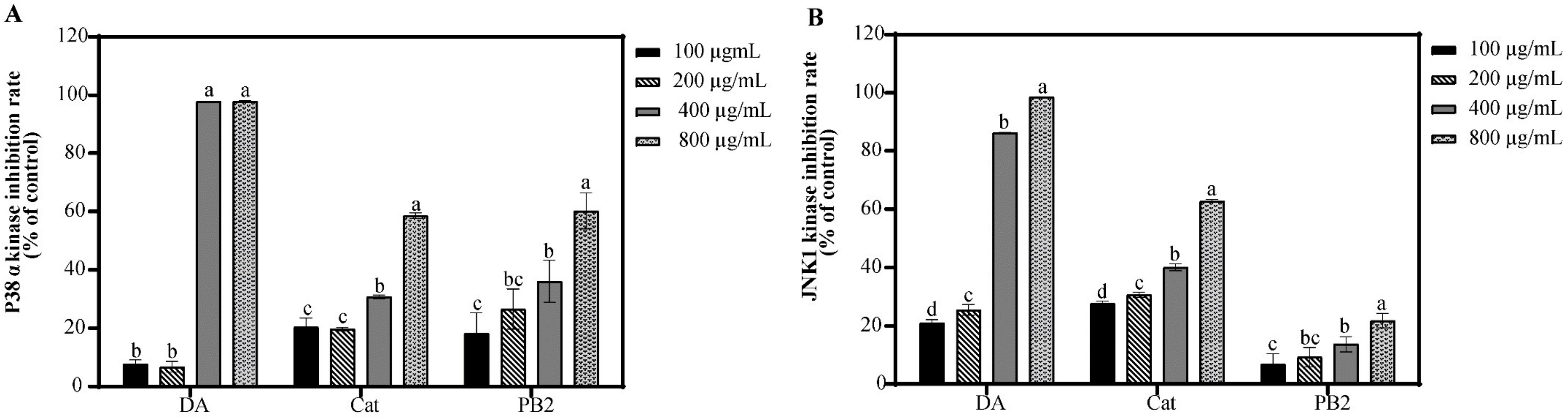

Figure S2. The inhibition rate of (A) p38α and (B) JNK1 kinase activities treated with different concentrations of 3,4-dihydroxybenzoic acid, (+)-catechin, and procyanidin B2. DA, Cat, and PB2 indicate 3,4-dihydroxybenzoic acid, (+)-catechin, and procyanidin B2, respectively. Different letters above the columns indicate statistically significant differences ($p < 0.05$). Results are presented as mean ± S.D. ($n = 3$).

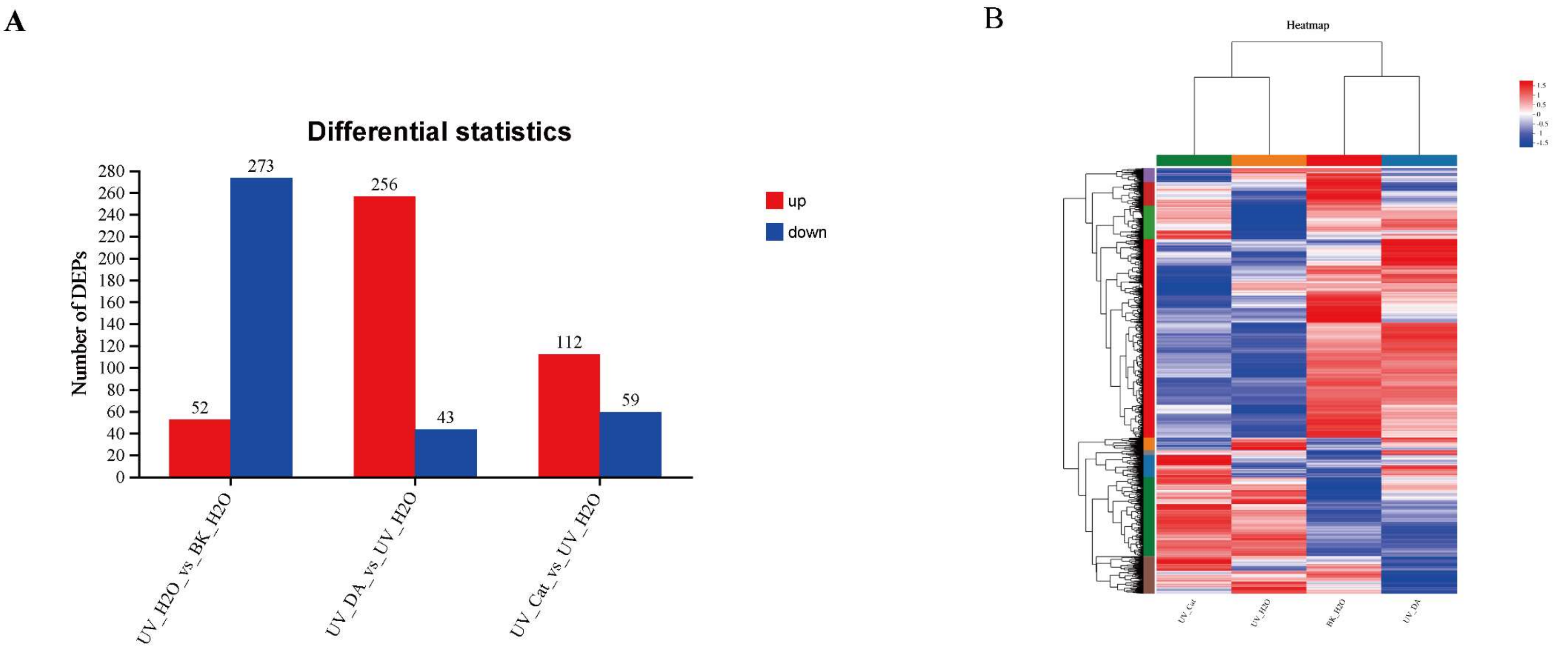

Figure S3. The statistics and heatmap cluster of differentially expressed proteins treated with 3,4-dihydroxybenzoic acid and (+)-catechin. UV_H2O and BK_H2O indicate HaCaT cells in the presence and absence of UVB exposure (225 mJ/cm²) after 12 h of cultivation, respectively. UV_DA and UV_Cat indicate HaCaT cells treated with 200 µg/mL 3,4-dihydroxybenzoic acid and (+)-catechin after 12 h post-UVB treatment (225 mJ/cm²), respectively.

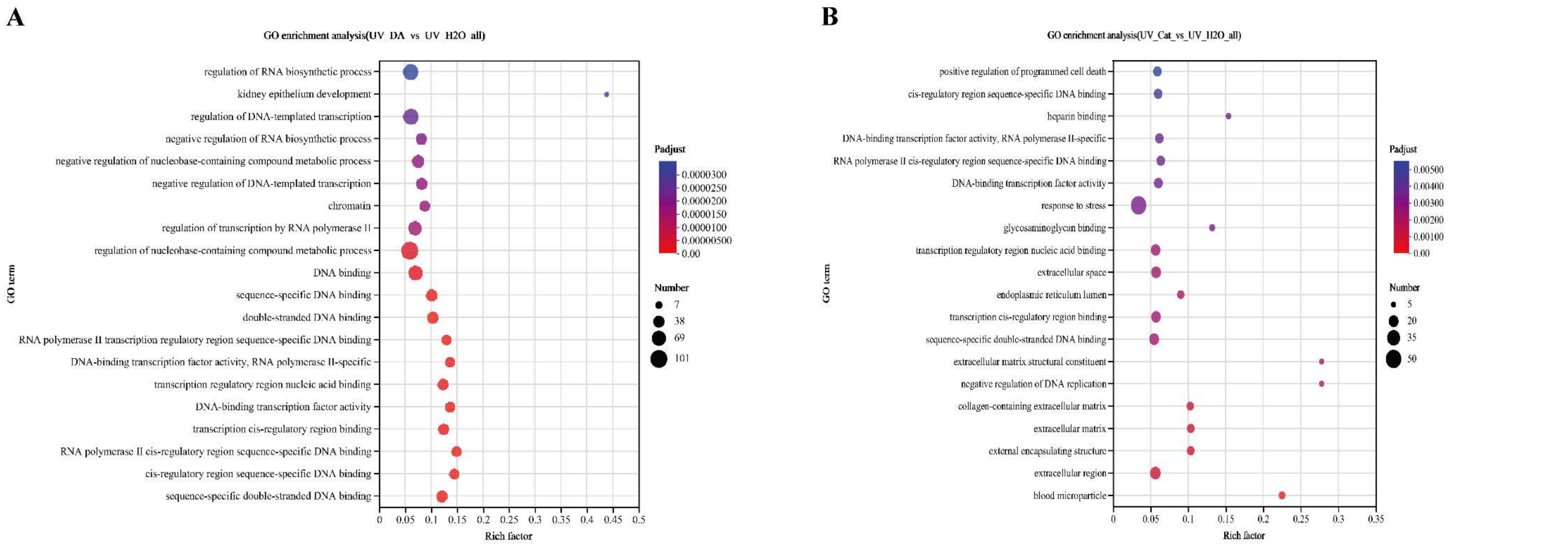

Figure S4. GO enrichment analysis of differentially expressed proteins treated with 3,4-dihydroxybenzoic acid and (+)-catechin after 12 h post-UVB treatment (225 mJ/cm²). UV_H2O indicates HaCaT cells in the presence of UVB exposure (225 mJ/cm²) after 12 h of cultivation. UV_DA and UV_Cat indicate HaCaT cells treated with 200 μg/mL 3,4-dihydroxybenzoic acid and (+)-catechin after 12 h post-UVB treatment (225 mJ/cm²), respectively.

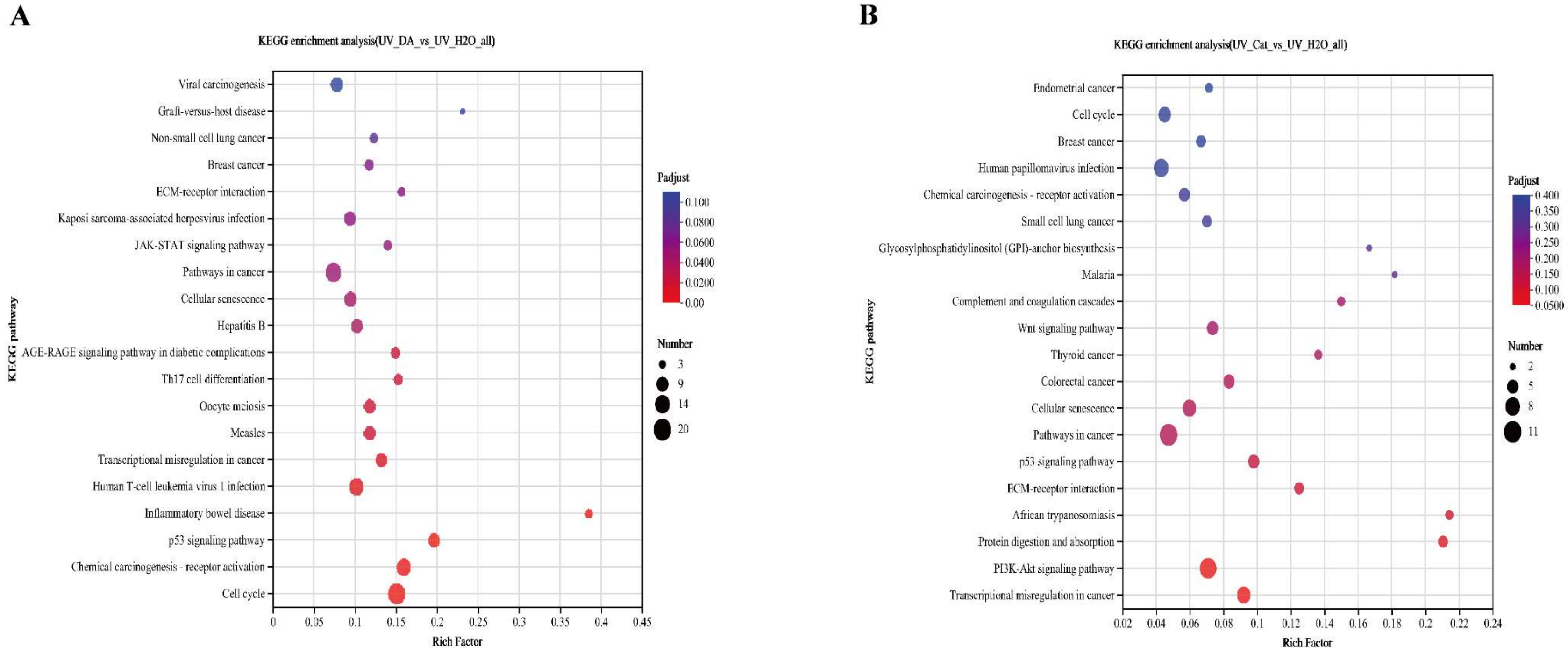

Figure S5. KEGG enrichment analysis of differentially expressed proteins treated with 3,4-dihydroxybenzoic acid and (+)-catechin after 12 h post-UVB treatment (225 mJ/cm²). UV_H2O indicates HaCaT cells in the presence of UVB exposure (225 mJ/cm²) after 12 h of cultivation. UV_DA and UV_Cat indicate HaCaT cells treated with 200 μg/mL 3,4-dihydroxybenzoic acid and (+)-catechin after 12 h post-UVB treatment (225 mJ/cm²), respectively.

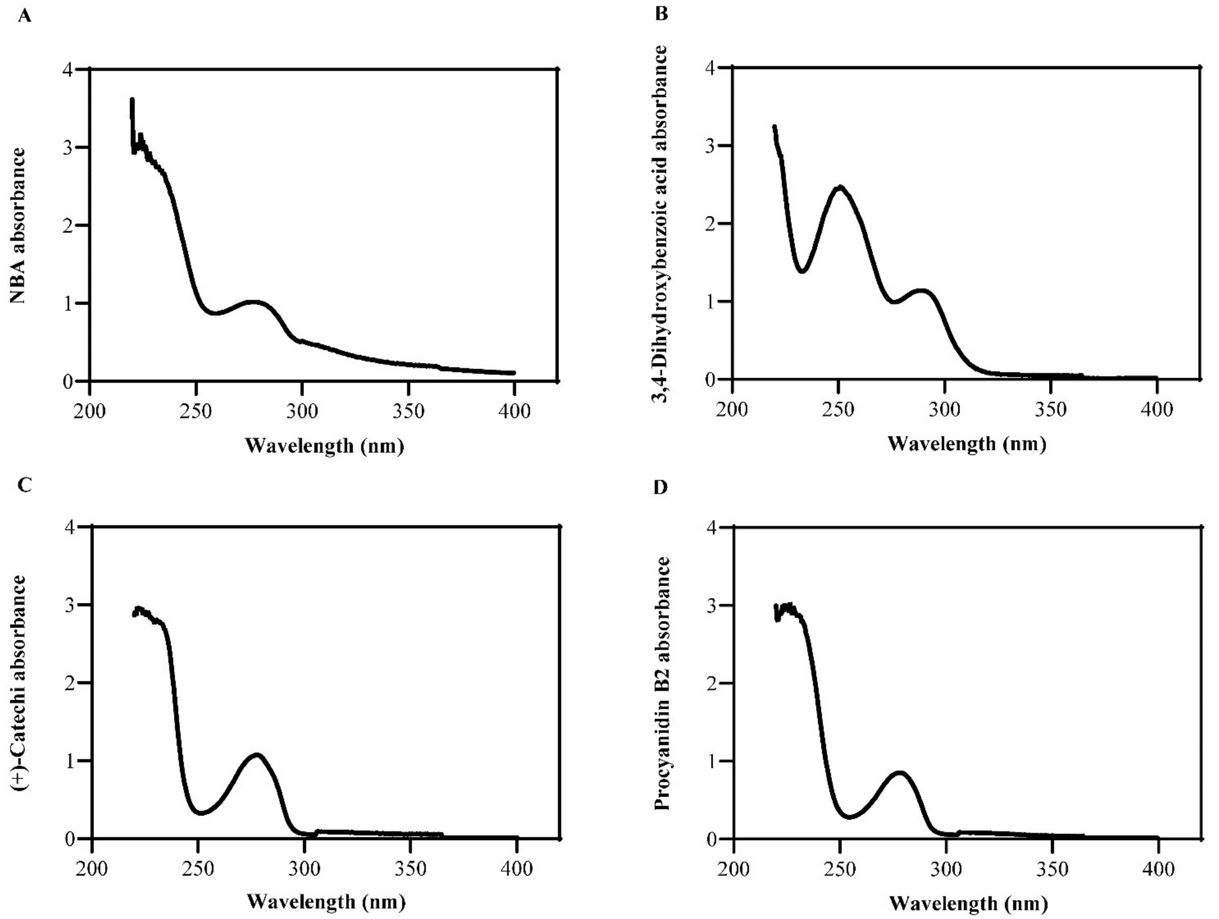

Figure S6. Analysis of the absorption wavelengths for (A) NBA, (B) 3,4-dihydroxybenzoic acid, (C) (+)-catechin, and (D) procyanidin B2, ranging from 220 nm to 400 nm.
